# Supplementary material for: Causal Relationship between Aging and Anorexia Nervosa: A White-Matter-Microstructure-Mediated Mendelian Randomization Analysis
Source: Biomedicines. 2024 Aug 16;12(8):1874. doi: 10.3390/biomedicines12081874 (PMC11351342; doi:10.3390/biomedicines12081874)
Supplement: Supplementary file 1 [file biomedicines-12-01874-s001.zip › Supplementary Figure/Figure S8/Leaveoneout plot of MR analysis for TL to ubm-b-1846.pdf]

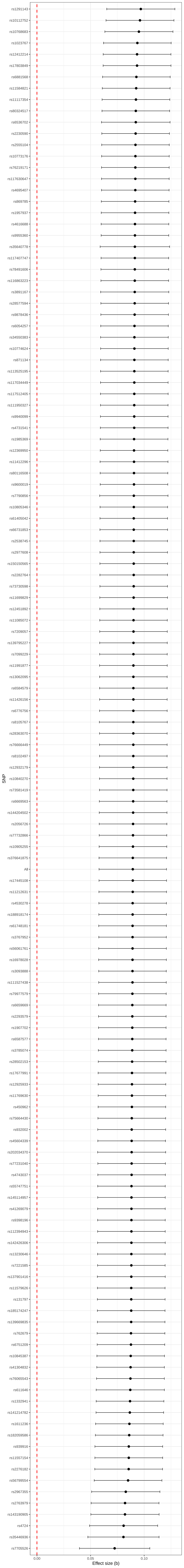
rs1291143
rs10112752
rs10768683
rs1023767
rs12412214
rs17803849
rs6881568
rs11584821
rs11117354
rs80324517
rs6536702
rs2230590
rs2555104
rs10773176
rs76219171
rs117630647
rs4695407
rs669785
rs1957937
rs4616688
rs9955360
rs35640778
rs117407747
rs78491606
rs116863223
rs3891167
rs28577594
rs9878436
rs6054257
rs34550383
rs10774624
rs871134
rs113525195
rs117034449
rs117512405
rs111950327
rs9940099
rs4731541
rs1985369
rs12369950
rs11412296
rs80116508
rs9600019
rs7790856
rs10805346
rs61405042
rs66731853
rs2538745
rs2977608
rs150150565
rs2282764
rs73730598
rs11699829
rs12451892
rs11085072
rs7209057
rs139795227
rs7099229
rs11991877
rs13062095
rs6584579
rs11426156
rs6776756
rs8105767
rs28363070
rs76666449
rs8102497
rs12932179
rs10840270
rs73581419
rs6669563
rs144204502
rs2056726
rs77732866
rs10905255
rs376641875
All
rs17445108
rs11212631
rs4530278
rs188918174
rs61748181
rs3767952
rs56061761
rs16978028
rs3093888
rs111527438
rs79977579
rs6659669
rs2293579
rs1907702
rs6587577
rs3785074
rs28502153
rs17677991
rs12925933
rs11769630
rs450962
rs75664430
rs932002
rs45604339
rs202034370
rs77231040
rs4743037
rs55747751
rs145114957
rs41269079
rs9398196
rs112394943
rs142426306
rs13230646
rs7221585
rs137901416
rs11579626
rs131797
rs185174247
rs139669835
rs762679
rs6751209
rs10845387
rs41304832
rs76065543
rs611646
rs1332941
rs141214782
rs1611236
rs182059586
rs939916
rs11557154
rs2276182
rs56799554
rs2967355
rs2763979
rs143190905
rs4724
rs35446936
rs7705526
SNP
0.00
0.05
0.10
Effect size (b)
